# Retinal and Choroidal Microvascular Changes Across the Migraine Attack Cycle: A Systematic Review and Exploratory Quantitative Synthesis

**DOI:** 10.7759/cureus.117330

**Published:** 2026-10-01

**Authors:** Abdullah Q Banimohammad, Yael Reingold, Zaid A Al-Samarat, Dina M Khliefat, Rama Raed Abu Zitar, Sameh N Alqawasmeh, Sarah A Alshamaly, Carole Graves, Maya Jabaji, Celine Aljazzar, Abdullah Anshassy, Mohammed M Al-Rashdan

**Affiliations:** 1 Neurosurgery, King Abdullah University Hospital, Irbid, JOR; 2 School of Medicine, International University of the Health Sciences, Basseterre, KNA; 3 School of Medicine, University of Jordan, Amman, JOR; 4 Faculty of Medicine, Mutah University, Amman, JOR; 5 School of Medicine, Columbia University, New York, USA; 6 Faculty of Medicine, Jordan University of Science and Technology, Irbid, JOR

**Keywords:** choriocapillaris, ictal, interictal, migraine, octa, optical coherence tomography angiography, retinal microvasculature

## Abstract

Migraine is an episodic neurovascular disorder, but whether ocular microvascular abnormalities detected by optical coherence tomography angiography (OCTA) vary across the attack cycle or represent persistent interictal characteristics remains uncertain.

This systematic review evaluated phase-anchored retinal, optic nerve head, and choroidal OCTA findings and assessed the consistency of available quantitative evidence. PubMed, Scopus, Web of Science, and Google Scholar were searched from inception through June 25, 2026, for original human studies reporting OCTA measurements during ictal, interictal, or clearly defined attack-free periods. Seven observational studies met the eligibility criteria, of which 3 (43%) provided paired ictal-interictal measurements; additional studies contributed ictal-versus-healthy-control and interictal-versus-control evidence, with some studies contributing to more than one comparison family. The strongest attack-related signal involved reduced parafoveal perfusion, including an approximately 7% decrease in vessel flux index during attacks in both migraine-with-aura and migraine-without-aura groups, together with a reduction in foveal choriocapillaris vessel density from 64.91% interictally to 63.34% ictally. Interictal-control studies also identified lower central superficial and deep capillary plexus vessel density and larger foveal avascular zone measurements in selected cohorts, whereas retinal and peripapillary findings overall were less consistent. After accounting for shared controls, same-study subgroups, paired measurements, bilateral-eye dependence, anatomical region, vascular layer, outcome metric, and measurement unit, no outcome stratum contained at least two independent statistically compatible studies suitable for formal pooling. Methodological concerns centered mainly on statistical analysis, bilateral-eye handling, confounding, and incomplete reporting, and certainty of evidence was very low. Overall, the findings support phase- and compartment-specific ocular microvascular changes in migraine, with the clearest dynamic abnormalities involving the parafoveal and choriocapillaris circulation. However, the current evidence remains insufficient to establish OCTA as a reproducible clinical or diagnostic biomarker.

## Introduction and background

Migraine is a recurrent neurological disorder involving complex interactions among neuronal, trigeminovascular, autonomic, sensory, and vascular mechanisms. Because patients alternate between symptomatic attacks and relatively symptom-free intervals, vascular measurements obtained during headache may represent transient physiological changes, whereas abnormalities persisting between attacks may reflect more stable characteristics.

The retina and choroid provide accessible vascular beds for non-invasive investigation of these changes. OCTA visualizes perfused vessels without intravenous contrast and permits quantitative assessment of the superficial capillary plexus (SCP), deep capillary plexus (DCP), radial peripapillary capillaries (RPC), foveal avascular zone (FAZ), optic nerve head circulation, and choriocapillaris. Previous OCTA studies have demonstrated retinal and choroidal differences between patients with migraine and healthy controls [1-4]. However, many were cross-sectional or did not clearly relate imaging to the timing of migraine attacks.

Phase-anchored studies can better distinguish transient attack-related abnormalities from persistent vascular characteristics. Repeated ictal-interictal imaging is particularly informative because each participant serves as their own comparator. Interpretation remains challenging; however, because available studies differ in OCTA platform, scan dimensions, segmentation procedures, vascular compartments, outcome metrics, migraine phenotype, and treatment of bilateral-eye measurements [5-11]. An important unresolved question is whether reported ocular microvascular abnormalities represent transient ictal physiology or persistent interictal characteristics. Retinal and choroidal vascular measurements may provide a non-invasive window into migraine-related vascular regulation, although OCTA does not directly measure cerebral perfusion.

The present review therefore focused specifically on phase-anchored evidence, evaluating how retinal, optic nerve head, and choroidal OCTA measurements differ across defined ictal, interictal, and attack-free periods and whether the available data are sufficiently comparable for quantitative synthesis. Operationally, the review considered participants with migraine, OCTA acquisition during a defined attack-cycle phase, an alternative migraine phase or healthy-control comparator, and OCTA-derived retinal, optic nerve head, or choroidal microvascular outcomes. Methodological quality and certainty of evidence were also assessed.

## Review

Methodology

Literature Search

This systematic review was conducted in accordance with the Preferred Reporting Items for Systematic Reviews and Meta-Analyses (PRISMA) 2020 statement [12]. The review protocol and related materials were made publicly available on the Open Science Framework (OSF) [13]. PubMed and Scopus were searched on June 23, 2026, Web of Science on June 24-25, 2026, and Google Scholar on June 25, 2026; therefore, the final search date was June 25, 2026. The PubMed strategy was (migraine OR “migraine with aura” OR “migraine without aura” OR “retinal migraine” OR “ocular migraine”) AND (“OCT angiography” OR OCTA OR “optical coherence tomography angiography”) AND (“vessel density” OR “retinal microvasculature” OR “retinal perfusion” OR “superficial capillary plexus” OR “deep capillary plexus” OR “foveal avascular zone” OR FAZ). Scopus and Web of Science were searched using migraine AND OCTA; 47 records were retrieved from Scopus, and no records were retrieved from Web of Science. Google Scholar was searched using “migraine” AND “optical coherence tomography angiography” and was used as a supplementary rather than primary bibliographic source. Because Google Scholar relies on a dynamically updated, relevance-ranked algorithm rather than a fixed reproducible bibliographic index, a fixed screening window of the first 50 relevance-ranked records was predefined to standardize the supplementary search and provide a reproducible stopping rule. Eighteen records were imported from Google Scholar. Search results were managed and deduplicated using Zotero.

Eligibility Criteria and Study Selection

Original human studies were eligible if they enrolled participants with migraine, used OCTA to evaluate retinal, optic nerve head, or choroidal microvascular outcomes, and documented imaging during an ictal, interictal, headache-free, or otherwise clearly defined attack-cycle phase. Both repeated-measures studies and phase-defined comparisons with headache-free controls were eligible. Ictal status was defined according to the primary study as OCTA acquisition during an active migraine attack. Interictal or attack-free status required a study-defined symptom-free interval surrounding OCTA acquisition. Because phase definitions varied among the included studies, a universal interval was not imposed. Studies were excluded if they did not use OCTA, lacked adequately defined migraine-phase timing, did not provide migraine-specific vascular outcomes, or represented duplicate or overlapping reports without additional independent data. Reviews, editorials, protocols, non-human studies, and structural OCT studies without eligible OCTA-derived microvascular measurements were also excluded. Two reviewers assessed eligibility, with disagreements resolved through discussion.

Data Extraction

Data were extracted using a standardized framework and included study design, country, sample size, migraine phenotype, aura status, attack-phase definition, comparator, participant and eye counts, OCTA platform, scan dimensions, segmentation, anatomical region, vascular layer, outcome metric, numerical findings, and statistical methods. Participant- and eye-level observations were distinguished because bilateral measurements from the same participant are correlated. For repeated ictal-interictal studies, phase-specific means and standard deviations were extracted where available, together with the standard deviation of within-participant change or within-person correlation when reported.

During revision, all quantitative fields used in the synthesis were re-audited against the corresponding primary-study tables and full texts. Comparison families were treated as non-mutually exclusive because one study could contribute to more than one comparison type. Study independence was therefore evaluated separately for each extracted effect rather than inferred from the number of subgroups or extracted rows. The analytical unit was classified as participant-level, single-eye, bilateral-eye, or paired repeated measurement. Newly calculated conventional confidence intervals were not generated from bilateral-eye summaries when the source analysis did not adequately account for within-participant eye correlation. Data extraction was performed independently by two reviewers, and any discrepancies were resolved through discussion and consensus.

Risk-of-Bias Assessment

Risk of bias was appraised using design-appropriate Joanna Briggs Institute (JBI) critical appraisal principles [14]. Assessment considered eligibility and selection, participants and setting, migraine-phase ascertainment, OCTA outcome measurement, confounding control, statistical analysis, bilateral-eye unit of analysis, and completeness of reporting. Domain judgments were categorized as low concern, some concern, or high concern. These categories represent review-specific summaries informed by JBI principles rather than numerical JBI scores. No composite overall study-level risk-of-bias category was calculated.

Certainty was additionally summarized at the outcome-family level using a GRADE-style framework considering risk of bias, inconsistency, indirectness, imprecision, and independent replication. Given the observational designs, limited evidence base, methodological heterogeneity, and lack of replication of specific OCTA outcomes, certainty was interpreted conservatively.

Statistical Analysis

Continuous OCTA outcomes were summarized as raw mean differences (MDs) when measurements were reported on the same scale. Effect direction was defined as the migraine or ictal condition minus its comparator; negative values therefore generally indicate lower measurements in the migraine or ictal condition. Clinical and statistical compatibility was assessed according to comparison family, anatomical region, vascular layer, OCTA metric, measurement unit, paired structure, analytical unit, and study independence. SCP, DCP, RPC, FAZ, choriocapillaris, flow area, perfusion density, and vessel flux index were not treated as interchangeable outcomes.

For selected independent participant-level comparisons with sufficient means, standard deviations, and sample sizes, descriptive Welch-based 95% confidence intervals around raw MDs were calculated to illustrate uncertainty. These review-derived intervals were not used to replace inferential testing reported by the primary studies. For paired ictal-interictal comparisons, a confidence interval for within-participant change was not reconstructed unless the standard deviation of change, within-person correlation, or individual paired observations were available. For studies reporting both eyes without adequate adjustment for within-person correlation, source-reported p-values and descriptive point estimates were retained without newly calculated conventional confidence intervals. Source-reported adjusted effects were retained on their original scale.

Formal pooling required at least two independent studies with sufficiently compatible comparison type, anatomical region, vascular layer, outcome metric, measurement unit, and analytical structure. Following re-evaluation of shared controls, paired structure, bilateral-eye dependence, and study independence, no outcome stratum fulfilled these criteria. Accordingly, no formal pooled meta-analysis, I², τ², Q statistic, assumed-correlation sensitivity analysis, meta-regression, funnel plot, or publication-bias analysis was performed. The statistical workflow was implemented in R version 4.6.1, with the metafor package retained within the reproducible analytical environment [15]. Quantitative findings were ultimately presented as a descriptive study-level synthesis. The supplementary workbook contains the extracted summary statistics, analytical units, effect calculations, risk-of-bias judgments, source discrepancies, and study-level quantitative data.

Results

Study Selection

The database searches contributed 96 records: 31 from PubMed, 47 from Scopus, and 18 from Google Scholar. Thirty-one duplicates were removed, leaving 65 unique records for title-and-abstract screening. Forty-eight records were excluded during title-and-abstract screening, and 17 reports underwent full-text assessment. Ten full-text reports were excluded from independent study inclusion: six because migraine-phase timing was inadequately defined, three because they evaluated structural OCT rather than eligible OCTA-derived microvascular outcomes, and one because it represented an overlapping companion report without additional independent data. Seven unique studies were included in the systematic review [5-11]. The companion report related to the Patzkó cohort was not counted as an independent study but was used to clarify interictal imaging timing for the corresponding published cohort. The study-selection process is shown in Figure 1.

**Figure 1:**
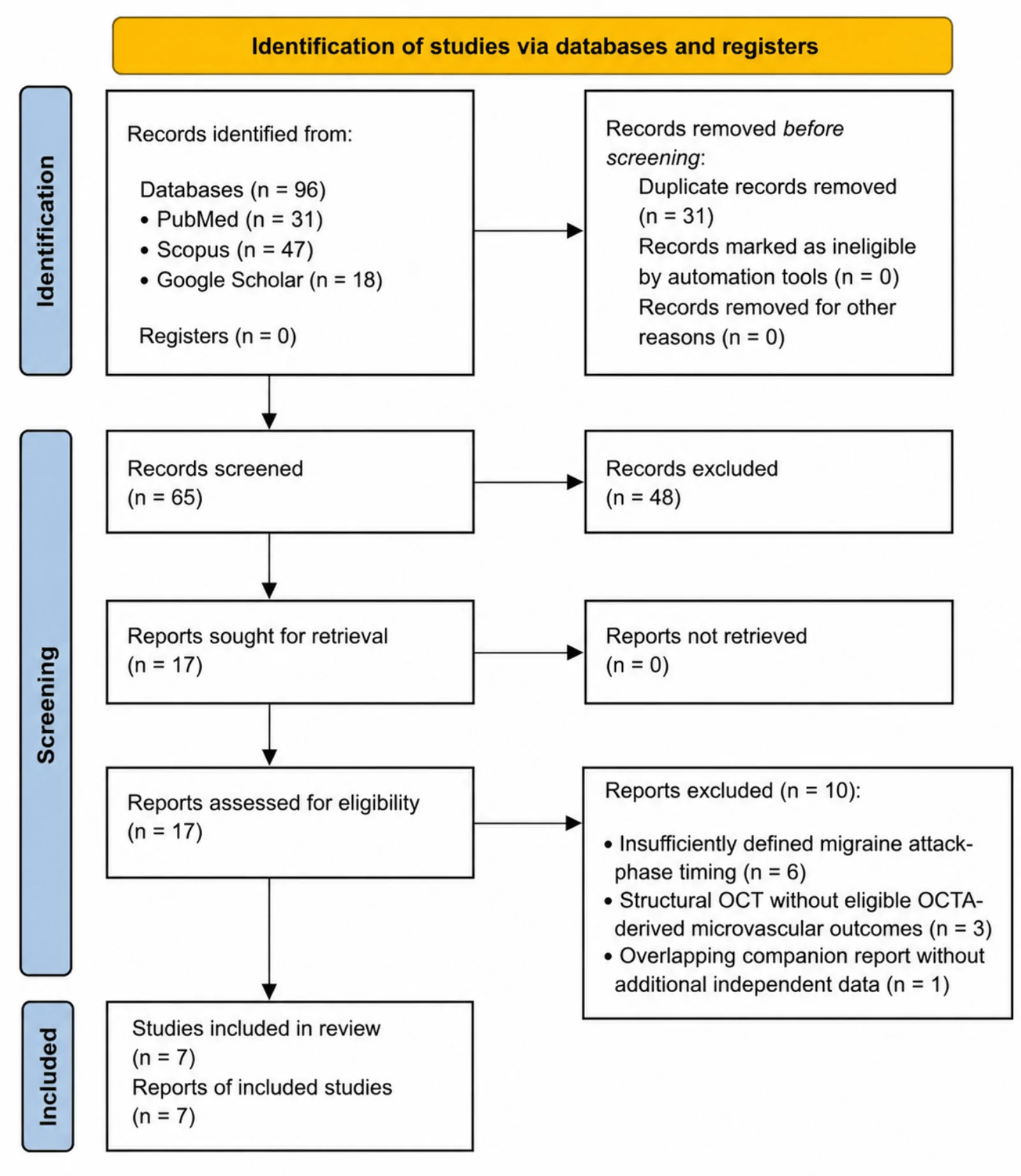
PRISMA 2020 flow diagram of study selection

Study Characteristics

Seven observational studies from Turkey, Hungary, the United States, Italy, and Brazil were included [5-11]. Comparison categories were not mutually exclusive because individual studies could contribute to more than one comparison family. Three studies provided paired ictal-interictal measurements [5-7]. Romozzi et al. and Güler et al. contributed ictal-versus-healthy-control comparisons [7,11], while Podraza et al. incorporated healthy controls and repeat control scans within their attack-related analyses [6]. Patzkó et al., Gürpınar and Süllü, and de Oliveira et al. provided the principal interictal or attack-free control comparisons [8-10]. Estimates originating from the same study or from subgroups sharing a common control group were not treated as independent studies for quantitative synthesis. The main characteristics of the included studies, including their phase definitions and analytical samples, are summarized in Table 1.

**Table 1:** Characteristics of included studies OCTA: optical coherence tomography angiography; MA: migraine with aura; MO: migraine without aura; SCP: superficial capillary plexus; DCP: deep capillary plexus; RPC: radial peripapillary capillaries; FAZ: foveal avascular zone

Study	Design and sample	Phase/comparison	Principal OCTA outcomes
Ekmekyapar Fırat et al. [5]	Paired repeated measures; 31 recruited, 27 completed paired imaging; 54 eyes	Ictal during untreated migraine attack; interictal after ≥7 symptom-free days	RPC vessel density
Podraza et al. [6]	Prospective repeated imaging; 37 MA, 30 MO, 20 healthy controls; ictal-return subset 12 MA, 8 MO; five controls had repeat scans	Interictal ≥24 h symptom-free; ictal during active migraine attack; healthy/repeat controls	Vessel flux and related vascular indices
Romozzi et al. [7]	Case-crossover; 13 migraine participants (26 eyes), 15 controls (30 eyes)	Untreated ictal attack ≤24 h from onset; interictal no migraine 72 h before and after imaging; ictal vs controls	FAZ, macular, disc, peripapillary, choriocapillaris
Patzkó et al. [8]	Observational case-control; 40 migraine participants recruited; 30 with a dominant side analyzed vs 25 controls	Interictal/attack-free vs controls; dominant vs non-dominant side	SCP, DCP, FAZ, optic nerve head
Gürpınar and Süllü [9]	Prospective case-control; 56 migraine participants, 51 controls; 112 and 102 eyes, respectively	Interictal ≥24 h after last attack and ≥24 h before next expected attack	SCP/DCP density, FAZ, vascular metrics
de Oliveira et al. [10]	Cross-sectional case-control; 31 migraine participants, 16 controls	Interictal ≥48 h without migraine attack	Superficial macular vessel density
Güler et al. [11]	Prospective case-control; 26 migraine participants (52 eyes), 24 controls (48 eyes)	Ictal; mean 2.0±1.24 h after attack onset; before acute treatment	SCP/DCP density, choriocapillaris and peripapillary flow

Risk of Bias

Risk-of-bias findings are presented at the domain level rather than as composite study-level ratings. Eligibility/selection, participant characterization, phase ascertainment, and OCTA outcome measurement were generally judged to have low concern. The principal concerns involved confounding, statistical analysis, bilateral-eye unit of analysis, and completeness of reporting. Across the 56 domain-level judgments, 39 (69.6%) were rated as low concern, 11 (19.6%) as some concern, and six (10.7%) as high concern. High-concern judgments were confined to the statistical-analysis and bilateral-eye domains in studies in which both eyes were analyzed without an explicit within-participant correlation model.

All five assessed outcome families were rated as having very low certainty. Downgrading reflected the small observational evidence base, absence of independent replication for specific outcomes, methodological heterogeneity, incomplete paired-variance reporting, and analytical-unit concerns. The study-level domain judgments and their distribution across the evidence base are shown in Figures 2, 3.

**Figure 2:**
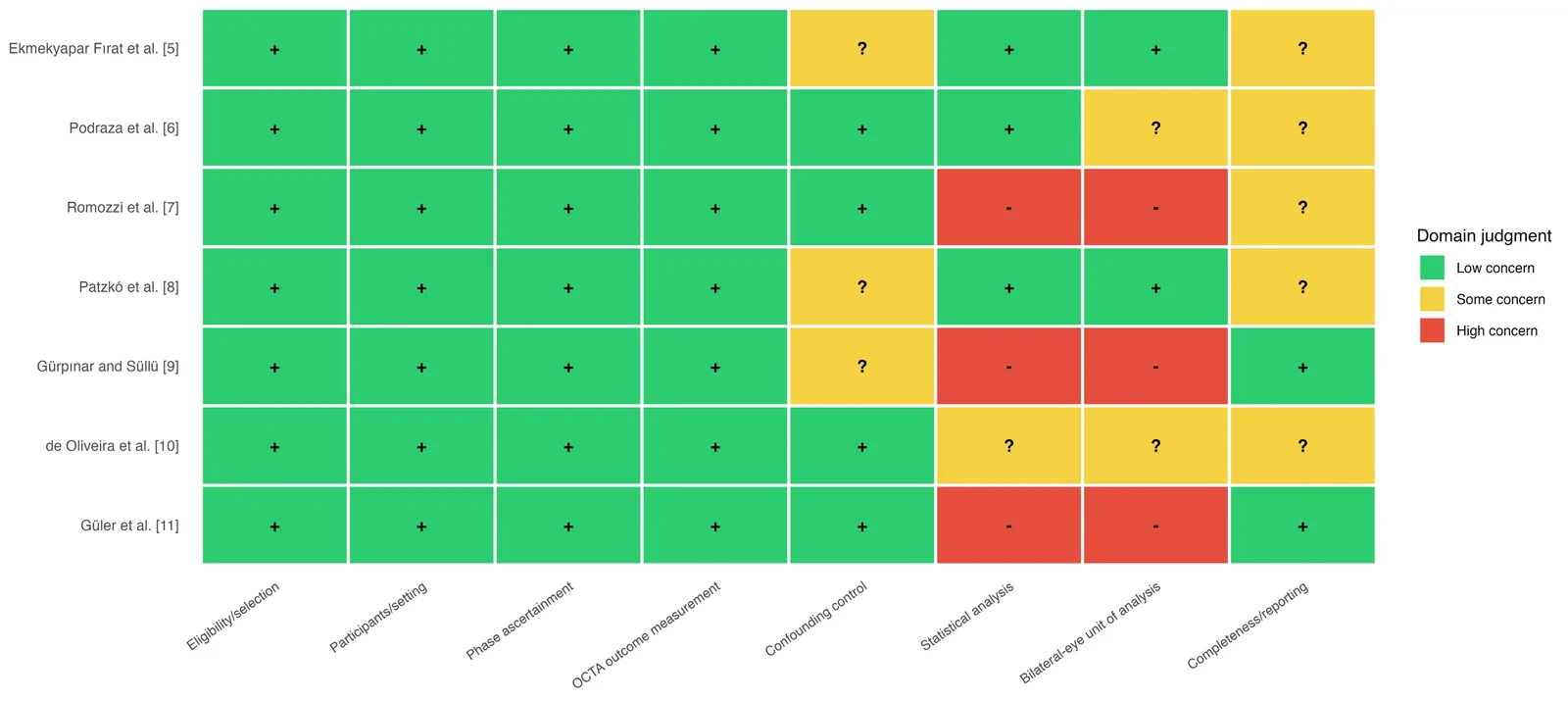
Domain-level risk-of-bias appraisal of included studies Green indicates low concern, yellow indicates some concern, and red indicates high concern. Judgments represent domain-level methodological assessments informed by design-appropriate JBI principles. No numerical JBI score or composite overall study rating was assigned.

**Figure 3:**
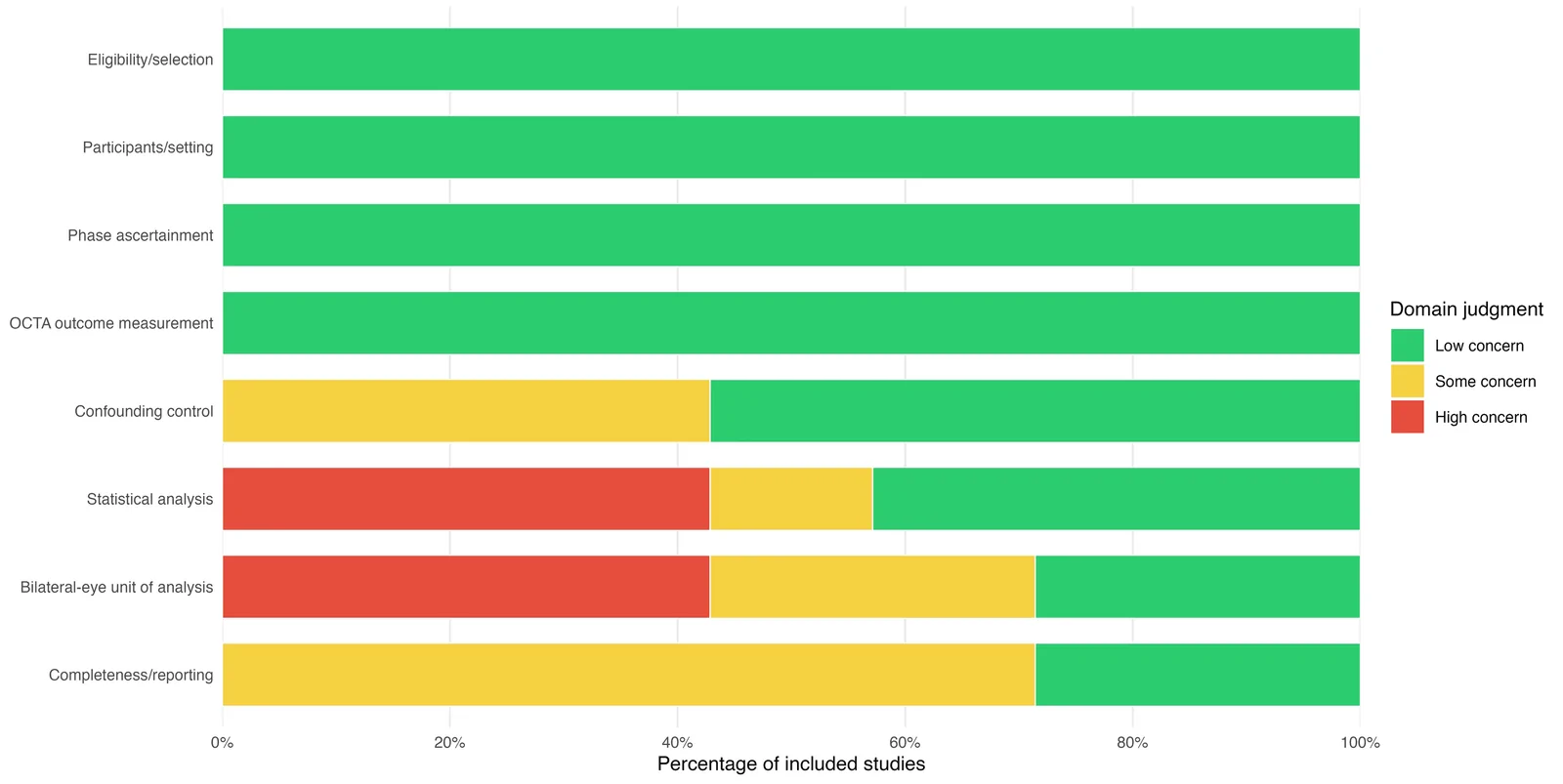
Summary of risk-of-bias judgments across included studies Percentages summarize the distribution of low-, some-, and high-concern judgments across the eight assessed methodological domains for the seven included studies [5-11]. Percentages should not be interpreted as overall study-level ratings.

OCTA Findings Across the Migraine Attack Cycle

Three studies provided direct paired ictal-interictal measurements [5-7]. Ekmekyapar Fırat et al. reported small RPC vessel-density changes, with descriptive MDs of approximately −0.44 to 0.00 percentage points and no clear attack-related RPC change [5]. Podraza et al. reported an approximately 7% reduction in parafoveal vessel flux index during attacks in both migraine with aura (95% CI, −10% to −4%; p=0.006) and migraine without aura (95% CI, −10% to −3%; p=0.016), compared with a +2% repeat-scan change in healthy controls (95% CI, −3% to 7%) [6]. Romozzi et al. reported lower foveal choriocapillaris vessel density ictally than interictally, decreasing from 64.91% to 63.34% (MD −1.57 percentage points) [7]. The source table reports p=0.019, whereas the narrative reports p=0.0019; the tabulated value was retained. Because paired variance was unavailable for the relevant Fırat and Romozzi outcomes, review-derived paired confidence intervals were not calculated.

Two studies contributed direct ictal-versus-healthy-control evidence [7,11]. Romozzi et al. reported a larger FAZ area and lower inside-disc, foveal, and foveal choriocapillaris vessel density during attacks [7]. Güler et al. reported a superior DCP vessel-density MD of −1.81 percentage points, based on 61.93±4.90% during attacks versus 63.74±3.26% in controls, with a source-reported p=0.050 [11]. The previously reconstructed confidence interval was removed because both eyes were analyzed without an explicit clustering model. This finding was therefore retained as a potentially relevant ictal-control signal but not interpreted as a precise independently replicated effect.

Interictal-control findings were heterogeneous. Patzkó et al. reported lower central SCP vessel density (MD −2.62 percentage points), lower central DCP vessel density (MD −3.50 percentage points), and a larger FAZ area (MD +60.24 µm²) [8]. Gürpınar and Süllü reported lower foveal SCP and DCP vessel density, particularly among participants with migraine with aura [9]. Because their core vessel-density analyses used bilateral-eye data without an explicit within-person correlation model, newly calculated conventional confidence intervals were not generated. De Oliveira et al. reported comparatively small superficial macular vessel-density differences [10]; these findings were retained descriptively because the reported analytical unit and group testing were not sufficiently clear for reconstruction of pairwise precision estimates. The principal study-level quantitative findings and their interpretation are summarized in Table 2.

**Table 2:** Quantitative synthesis summary

Study	Principal finding	Interpretation
Ekmekyapar Fırat et al. [5]	RPC MDs approximately −0.44 to 0.00 percentage points	No clear paired ictal RPC change
Podraza et al. [6]	Parafoveal VFI: MA −7% (95% CI −10% to −4%); MO −7% (95% CI −10% to −3%); control repeat +2% (95% CI −3% to 7%)	Source-reported attack-related perfusion reduction; VFI is distinct from conventional VD
Romozzi et al. [7]	Paired choriocapillaris MD −1.57 percentage points; source-table p=0.019; separate ictal-control abnormalities	Paired choroidal change plus separate ictal-control evidence
Patzkó et al. [8]	Central SCP MD −2.62; central DCP MD −3.50; FAZ MD +60.24 µm²	Selected interictal/control differences
Gürpınar and Süllü [9]	Lower foveal SCP/DCP, most prominent with aura	Phenotype-specific interictal differences; bilateral-eye precision uncertain
de Oliveira et al. [10]	Small superficial macular vessel-density differences	Limited interictal signal; analytical-unit ambiguity
Güler et al. [11]	Superior DCP MD −1.81 percentage points; source p=0.050	Ictal/control signal; conventional review-derived CI omitted because of bilateral-eye dependence

Exploratory Quantitative Synthesis

Quantitative compatibility was re-evaluated according to comparison family, anatomical region, vascular layer, metric, unit, paired structure, analytical unit, and study independence. After accounting for shared controls and same-study subgroup estimates, no exact outcome stratum contained at least two independent statistically compatible studies.

The four apparent two-study strata in the original analysis were based on subgroup estimates from the same study and/or a shared control group rather than two independent studies and therefore did not satisfy the requirements for formal pooling. A formal meta-analysis was consequently not performed in the revised synthesis. Study-level quantitative estimates were instead presented descriptively while preserving comparison family and measurement unit. Confidence intervals were shown only when directly reported or appropriately derived for descriptive purposes from independent participant-level data. Estimates affected by unavailable paired variance or unresolved bilateral-eye dependence were presented without newly reconstructed conventional confidence intervals. These estimates are displayed in Figure 4.

**Figure 4:**
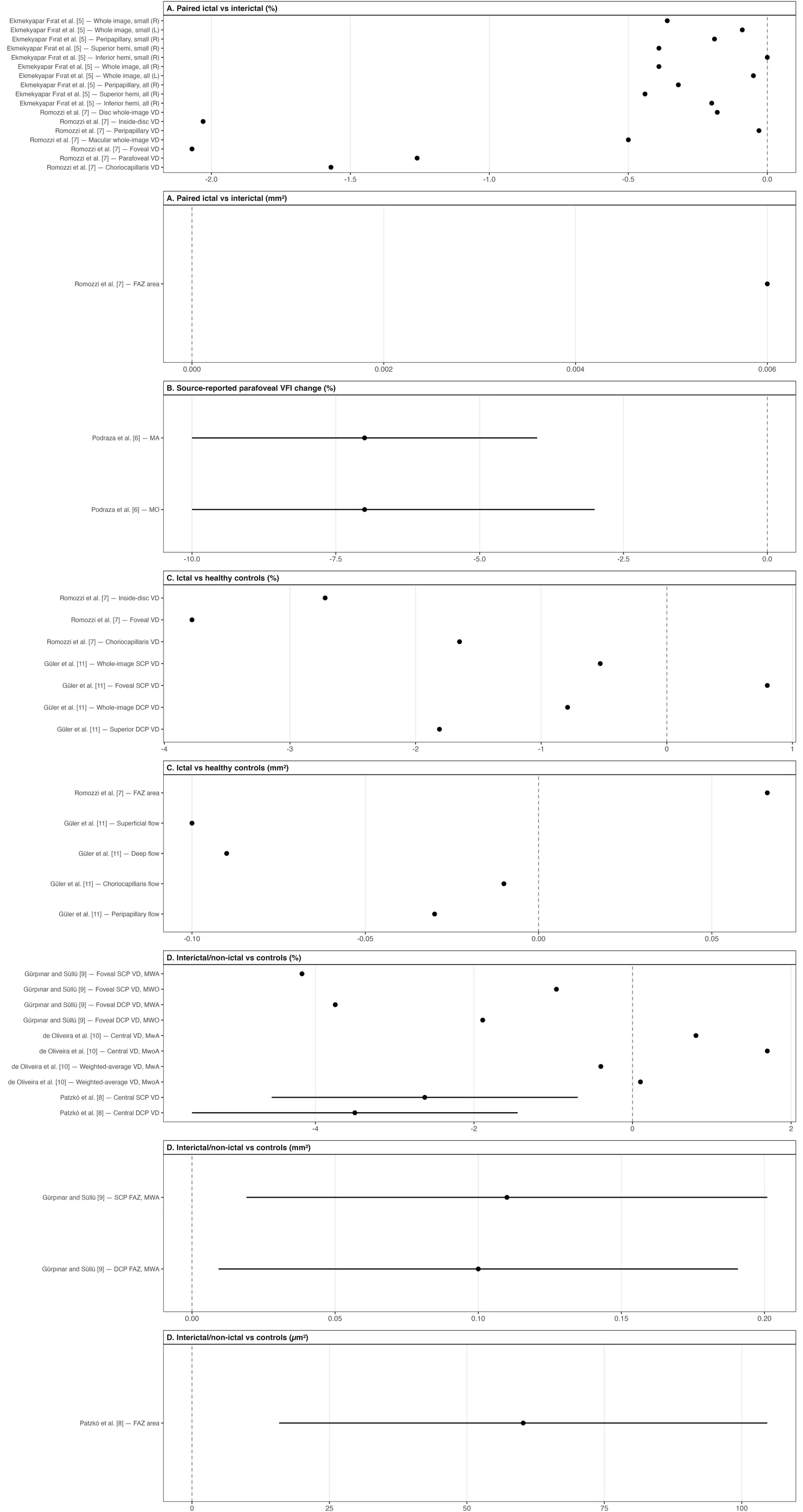
Study-level quantitative OCTA findings across migraine phases Study-level estimates are separated by comparison family and measurement unit. Panel A presents paired ictal-versus-interictal findings; Panel B presents source-reported parafoveal vessel flux index changes; Panel C presents ictal-versus-healthy-control comparisons; and Panel D presents interictal/non-ictal-versus-control comparisons. Points represent study-level estimates, horizontal lines indicate valid 95% confidence intervals where available, and the dashed vertical line indicates the null. No pooled summary estimate is presented.

Discussion

The available evidence suggests that migraine-related ocular microvascular abnormalities vary according to both attack phase and vascular compartment. The clearest within-participant dynamic findings were an approximately 7% reduction in parafoveal vessel flux index and lower foveal choriocapillaris vessel density during attacks [6,7], whereas RPC changes were small and inconsistent [5]. These paired findings should be distinguished from ictal-versus-control observations: Romozzi et al. reported additional retinal and choroidal abnormalities during attacks, while Güler et al. reported a superior DCP difference with a source p-value of 0.050 [7,11]. The latter was not grouped with the clearest dynamic findings because it represents an independent ictal-control comparison rather than a within-participant phase change and because its precision is additionally limited by bilateral-eye dependence. Interictal-control studies also identified selected SCP, DCP, and FAZ abnormalities, particularly in migraine with aura, but findings differed by anatomy, phenotype, OCTA platform, and analytical approach [8-10]. This heterogeneity is clinically relevant because vessel density, vessel flux, FAZ dimensions, retinal perfusion, and choriocapillaris measurements reflect different vascular properties and should not be interpreted as a single ocular microvascular outcome.

Migraine is clinically classified according to the International Classification of Headache Disorders [16], represents a major neurological and public-health burden [17], and remains an active area of biomarker and precision-medicine research [18]. Its pathophysiology also varies across different phases of the migraine cycle [19], providing biological justification for distinguishing ictal from interictal OCTA findings. OCTA offers non-invasive visualization of retinal and choroidal microvasculature, but its quantitative measurements are sensitive to acquisition platform, scan dimensions, segmentation, signal quality, image processing, and reporting methodology [20,21].

Representative previous OCT/OCTA reviews and meta-analyses have reported retinal microvascular abnormalities in migraine, including reductions in vascular or perfusion density and alterations in FAZ measures, while emphasizing substantial methodological heterogeneity [22-24]. Additional OCTA case-control evidence in migraine with aura has also identified retinal and optic nerve head vascular differences [25]. The present review extends this literature by organizing evidence primarily according to attack phase and by explicitly separating paired ictal-interictal changes from independent ictal-control and interictal-control comparisons.

The observed parafoveal and choroidal changes are biologically compatible with dynamic neurovascular or vasomotor alterations during migraine attacks and may generate hypotheses concerning attack physiology, imaging biomarkers, and treatment-response monitoring. Their clinical significance remains uncertain, however, because no included study established a validated diagnostic threshold, clinically meaningful minimum change, or reproducible association with attack severity or treatment response. Medication exposure and vascular risk also differed between studies and may influence ictal OCTA measurements. Current evidence therefore does not support OCTA for migraine diagnosis, determination of attack phase, or treatment guidance.

The revised quantitative assessment demonstrates why formal pooling is not currently appropriate. Once study independence, shared controls, paired structure, bilateral-eye dependence, anatomy, vascular layer, metric, and measurement unit were considered together, no individual OCTA outcome had independent statistically compatible replication. This does not indicate absence of a biological effect; rather, its magnitude and reproducibility cannot yet be estimated reliably. The main limitations are the small number of phase-defined observational studies, substantial clinical and methodological heterogeneity, incomplete paired-variance reporting, and inadequate handling of bilateral-eye dependence in several studies. Comparison families were overlapping rather than mutually exclusive, and the fixed first-50 Google Scholar window, although used to standardize a relevance-ranked supplementary search, may have reduced sensitivity for lower-ranked records. Future studies should use prospective paired designs, standardized phase definitions and OCTA acquisition, explicit treatment documentation, appropriate eye-level statistical models, and complete reporting of within-participant variance.

## Conclusions

Phase-anchored OCTA studies provide preliminary evidence that ocular microvascular physiology may vary across the migraine attack cycle, with the most distinct paired changes involving parafoveal vessel flux and choriocapillaris circulation. Ictal-control and interictal-control studies also identify retinal and choroidal abnormalities, but their magnitude and direction vary across vascular compartments, migraine phenotypes, imaging methods, and analytical approaches.

No individual OCTA outcome currently has sufficient independent and statistically compatible replication to support a reliable pooled effect estimate, and certainty of evidence remains very low. These findings may inform future investigation of migraine neurovascular mechanisms and candidate imaging markers of attack activity or treatment response, but no clinical or diagnostic application of OCTA is presently supported. Prospective paired studies using standardized phase definitions, OCTA protocols, medication documentation, appropriate bilateral-eye analysis, and complete paired-variance reporting are needed.
